# Clinical Application of Gait Retraining in the Injured Runner

**DOI:** 10.3390/jcm11216497

**Published:** 2022-11-01

**Authors:** Logan W. Gaudette, Molly M. Bradach, José Roberto de Souza Junior, Bryan Heiderscheit, Caleb D. Johnson, Joshua Posilkin, Mitchell J. Rauh, Lauren K. Sara, Lindsay Wasserman, Karsten Hollander, Adam S. Tenforde

**Affiliations:** 1 Spaulding Rehabilitation Hospital, Spaulding National Running Center, Department of Physical Medicine and Rehabilitation, Harvard Medical School, Boston, MA 02138, USA; 2 Graduate Program of Sciences and Technologies in Health, University of Brasilia, Brasilia 72220-275, DF, Brazil; 3 Department of Orthopedics and Rehabilitation, University of Wisconsin, Madison, WI 53706, USA; 4 United States Army Research Institute for Environmental Medicine, Military Performance Division, Natick, MA 01760, USA; 5 Doctor of Physical Therapy Program, San Diego State University, San Diego, CA 92182, USA; 6 Institute of Interdisciplinary Exercise Science and Sports Medicine, Faculty of Medicine, MSH, Medical School Hamburg, 20457 Hamburg, Germany

**Keywords:** gait retraining, running-related injuries, kinetics, kinematics, rehabilitation

## Abstract

Despite its positive influence on physical and mental wellbeing, running is associated with a high incidence of musculoskeletal injury. Potential modifiable risk factors for running-related injury have been identified, including running biomechanics. Gait retraining is used to address these biomechanical risk factors in injured runners. While recent systematic reviews of biomechanical risk factors for running-related injury and gait retraining have been conducted, there is a lack of information surrounding the translation of gait retraining for injured runners into clinical settings. Gait retraining studies in patients with patellofemoral pain syndrome have shown a decrease in pain and increase in functionality through increasing cadence, decreasing hip adduction, transitioning to a non-rearfoot strike pattern, increasing forward trunk lean, or a combination of some of these techniques. This literature suggests that gait retraining could be applied to the treatment of other injuries in runners, although there is limited evidence to support this specific to other running-related injuries. Components of successful gait retraining to treat injured runners with running-related injuries are presented.

## 1. Background

The sport of running has positive effects on both physical [1] and mental [2] wellbeing. Unfortunately, runners experience a high rate of running-related injuries (RRIs). While reports of incidence rate vary depending on the population, up to 79% of recreational runners suffer a RRI each year [3]. In addition, RRIs have a high rate of recurrence. For example, female youth runners with a history of bone stress injury (BSI) have a 5 times elevated risk of sustaining a subsequent BSI [4]. Similar rates of injury reoccurrence were found in high school cross country runners [5].

Prior research has characterized risk factors for RRI. Of those that are modifiable, risk factors include neuromuscular, kinetic, kinematic, and spatiotemporal variables. While muscle weakness and imbalance may contribute to RRIs [6], strengthening alone may be insufficient for modifying biomechanical abnormalities that contribute to RRI [7]. Several variables related to running mechanics are thought to be related to injury and are frequent targets of intervention, including hip adduction [8,9], trunk lean [10,11], vertical loading rates [12,13,14,15,16,17,18], and step rate [19,20,21,22].

Gait retraining has been described as a method to change running biomechanics contributing to a given RRI [23]. Gait retraining using external feedback was first described in patients following stroke [24]. It used concepts of motor learning for both acquisition and transfer phases and resulted in sustained improvements in hemiparetic gait. These concepts have since been applied to the management of RRIs by addressing a variety of aspects related to gait mechanics. Gait retraining typically involves the use of devices to measure the targeted biomechanical variable and provide external visual, verbal, or auditory cues to facilitate change. These external cues are described as biofeedback, and a faded biofeedback design refers to gradual reduction in external cues to promote learning of a new desired movement pattern without further feedback [24].

For gait retraining to be effective, biomechanical risk factors associated with RRI must be properly identified, and, if possible, addressed appropriately during treatment. The results of a recent systematic review and meta-analysis, which evaluated different forms of gait retraining [23], suggest that the literature surrounding gait retraining is occasionally inconsistent and largely inconclusive. There is need for a more easily digestible guide for clinicians seeking to implement gait retraining in the treatment of injured runners. The purpose of this narrative review is to provide a practical overview of what is known on biomechanical risk factors for RRI, gait retraining strategies to alter these risk factors, and provide clinical practical application of this knowledge.

## 2. Methods

This narrative review included studies related to the following topics: (I) Biomechanical risk factors for RRI, and (II) Gait retraining for runners with RRI. No restrictions were placed on language, publication date, participant age, gender, and duration of symptoms or stage of disease. Articles were excluded if: (I) running was not the primary focus of the study; (II) there was not a clear description of the gait retraining protocol used; (III) feedback was not removed after gait retraining to determine if gait adjustments could be maintained; (IV) the protocol did not use multiple sessions to allow for motor learning to occur. Additionally, studies that were not presented as a full manuscript (i.e., abstracts) were excluded. PubMed and EMBASE were the databases used. The date of the last search was 30 June 2022. Subject headings, synonyms, relevant terms, and variant spellings of three concepts (running biomechanics; gait retraining; running-related injuries) were used for the searches on each database. This strategy was used for each database with the appropriate truncation. All references were imported into Mendeley Reference Manager (Version 2.65.0), and duplicates were removed. The screening of eligible studies was performed in two steps: (I) screening the titles and abstracts, and (II) screening the full texts. List of references of the retrieved studies were searched to identify additional publications. Eligibility assessment was performed by two reviewers (LWG and MB). Disagreements were resolved by discussion between the two review authors. If no agreement could be reached, a final arbitration was performed by a third independent reviewer (AST). Relevant information was organized using the following topics: (I) Biomechanical risk factors for RRI; (II) Gait retraining overview; (III) Interventions characterizing gait retraining variables; (IV) Clinical application of gait retraining; (V) Limitation of current gait retraining strategies.

## 3. Biomechanical Risk Factors for Running-Related Injury (RRI)

Two recent systematic reviews of prospective studies have examined potential biomechanical risk factors for RRI [25,26]. Both reviews concluded that there was not strong evidence for a single biomechanical variable as a risk factor for all RRIs. The lack of an association between biomechanical variables and grouped RRI indicates the importance of investigating injury-specific biomechanical risk factors for RRI.

A recent systemic review examined biomechanical risk factors for several common RRIs including hamstring tendinopathy, patellofemoral pain syndrome (PFPS), patellar tendinopathy, iliotibial band syndrome, medial tibial stress syndrome, tibial stress fractures, Achilles tendinopathy, and plantar fasciitis [27]. Criteria for inclusion of a biomechanical risk factor in the study was a significant difference from a control group in one prospective study or two retrospective studies [27]. Levels of evidence for biomechanical risk factors for specific RRIs varied from conflicting evidence to moderate evidence.

The strongest evidence supported decreased braking impulse [28,29] and increased ground contact time [28,29] for PFPS, increased duration of rearfoot eversion angle [30,31] and increased contralateral pelvic drop angle [30,32] for medial tibial stress syndrome, and increased average [33,34] and instantaneous loading rate [33,35] of vertical ground reaction force for plantar fasciitis. Each biomechanical variable was observed to have moderate evidence as risk factors for specific RRIs. More limited evidence was found for biomechanical risk factors for Achilles tendinopathy and tibial stress fractures, and very limited evidence was found for iliotibial band syndrome. No biomechanical variables met the study criteria for inclusion for patellar tendinopathy and hamstring tendinopathy. Definitions of potential biomechanical risk factors can be found below in Table 1.

## 4. Gait Retraining Overview

A narrative review published in 2020 evaluated the use of gait retraining as an intervention for PFPS [37]. The review included mostly case series or studies that did not contain a control group. Most biomechanical interventions included in the review, specifically decreasing hip adduction, increasing trunk lean, transitioning from a rearfoot strike (RFS) to a forefoot strike pattern, and increasing cadence, resulted in a reduction of pain [37]. The review also concluded that greater gait retraining session volume and a faded feedback design resulted in better outcomes compared to studies without a faded feedback design.

While a previous systematic review conducted in 2015 found foot strike manipulation had the greatest effect on kinematic measures and live feedback of tibial acceleration had the greatest effect on kinetic measures [38], a more recent systematic review and meta-analysis of gait retraining by Doyle et al. [23], which included only randomized controlled trials, concluded that the best evidence for gait retraining for runners supported step rate-based gait retraining. Though it achieved only moderate evidence, step rate-based gait retraining was shown to increase step rate, decrease stride length, decrease peak hip adduction (HADD) during stance, increase footstrike angle at initial contact and decrease VALR and VILR. Moderate evidence was also found for the ability of tibial acceleration based gait retraining to lower VILR.

While some gait retraining studies have reported a decrease in pain and improvement in functional outcomes in patients that underwent gait retraining, an insufficient number of studies reported pain measurements or clinical outcomes compared to a control group for inclusion in the systematic review and meta-analysis for conclusions regarding the effectiveness of gait retraining on patient pain or clinical outcomes to be made [23]. A summary of gait retraining studies and their effect on various clinical and biomechanical outcome variables can be seen in Table 2.

## 5. Interventions Characterizing Gait Retraining Variables

Biomechanical targets of gait retraining studies (shown above in Table 2) include degree of hip adduction [8,9], step rate [19,20,21], tibial acceleration [12,14,15,16,17,18,39,45], footstrike pattern [41,42,43,44] and trunk lean [10]. The outcome measures of each study varied and included pain and functionality in injured runners, joint angles, and measures of loading rates (such as VALR and VILR) in healthy runners.

While the literature supporting hip adduction as a risk factor for PFPS is inconsistent [27], two studies have reported beneficial outcomes related to reduced hip adduction in runners with PFPS [8,9]. One study used a real time display generated by motion capture technology to display hip adduction [8], while the other study used visual feedback with a full-length mirror [9]. Both studies reported decreases in pain and increases in functionality, exceeding the minimal clinically important difference, at the end of the trial and at a 1- or 3-month follow-up.

Increases in step rate by 5% or 10% have been shown to lower COM vertical excursion, decrease breaking impulse and transition runners towards a more anterior footstrike pattern [36]. Three gait retraining studies have shown that step rate can be altered through gait retraining outside of a laboratory setting, including while runners continue with their training [20,21,50]. Willy et al. [20] found that this increase in step rate was also associated with a decrease in VALR, VILR and HADD [20]. A decrease in pain and increase in functionality was seen at the 6-month follow-up in runners that underwent gait retraining to increase their step rate by 10% [11]. The changes in step rate seen after gait retraining may not be constant at faster speeds, however [50]. One prospective study found no difference in KOS-ADLS scores between runners who received education on load management and underwent gait retraining based on step rate compared to those who only received education on load management [19]. A possible side effect of step rate-based gait retraining is calf muscle soreness. In one study, 43% of participants mentioned calf muscle soreness [49]. However, this did not affect running volume.

Tibial acceleration is a common variable of interest in gait retraining studies as a surrogate measure of loading rate. While a variety of techniques can be used to decrease tibial acceleration, some studies have found that runners are capable of lowering their tibial accelerations with visual feedback combined with instruction to land “softly” and “quietly” [14,39,41]. A study that used visual feedback of accelerometer data for gait retraining found significant reductions in tibial acceleration, VIP, VALR and VILR at post-training compared to pre-training [14]. Tibial acceleration, VIP, VALR and VILR all remained significantly reduced at 1-month, 6-month, and 12-month follow-ups.

Transitioning from a rearfoot strike to a forefoot strike has been shown to decrease loading rates while running [41]. Improvements in clinical outcomes that reach minimal clinically important difference in patients with PFPS that switch to a forefoot strike have been reported in a case series study [11]. These improvements remained at a 6-month follow-up. One randomized controlled trial found a significant reduction in pain in runners with PFPS that underwent gait retraining while the control group did not experience any significant changes in pain. However, subjects that underwent gait retraining reported calf soreness while undergoing gait retraining. Twenty-five percent of participants from the retraining group also reported ankle soreness at the 1-month follow-up when running more than 4 miles in a single session [42]. Chan et al. also found that runners who transitioned to a midfoot striking pattern did not display significantly different load rates compared to rearfoot strikers [43].

While fewer studies involving trunk lean were found, improvements in functional outcomes and decreases in pain were reported immediately upon the completion of gait retraining and at a 6-month follow-up [11]. Changes in functionality between pre-training and post-training reached minimal clinically important difference. These changes were similar to the changes seen in the treatment group that transitioned to a forefoot strike pattern.

## 6. Clinical Application of Gait Retraining

Sports injuries are complex and result from a combination of intrinsic and extrinsic factors [51]. Although biomechanics play a role in RRIs [27], it is important to highlight that a multifactorial perspective is required, and clinicians must identify the non-linear interactions between biomechanics and other aspects that may be related to injuries [51]. Gait retraining expands upon traditional approaches of addressing impairments in strength and flexibility to address abnormal biomechanics and motor control and should be considered as an aspect of a multimodal approach.

Most research on gait retraining strategies identified in this review (Table 2) focus on changing lower extremity mechanics to address knee pain. In PFPS, best evidence guidelines include exercises to improve hip and knee strength [52]. In the basics of the preliminary strengthening phase, the patient can also be considered in the cognitive stages of learning, using different exercises to gain intrinsic awareness of these muscles, and using visual cues to gain greater proprioceptive awareness. The treatment program then progresses to the associative stages of learning as tasks become more challenging. At this point, the participant should not only improve strength based on exercise prescription but improve ability to modify their alignment in a variety of tasks.

After addressing the pre-gait guidelines for management of PFPS, patients may further benefit by participating in gait retraining programs. Changes in pain and function up to six months were seen in runners with hip adduction greater than 20° that received visual feedback and were cued to “run with your knees apart with your kneecaps pointing straight ahead” [8,9], in runners with trunk flexion of 11° that were asked to “run with an increase in flexed trunk posture” [11], in runners with step rate between 160 to 170 steps/minute that were cued via an audio metronome set at 7.5–10% above their baseline step rate [11,19,49], and in runners that adopted a forefoot strike pattern using visual feedback and cues such as “run on your toes” and/or “run on the balls of your feet” [11,42].

The programs can be done using 8–12 sessions completed over 2–4 weeks (average of 2–3 gait retraining sessions per week). In this design, the feedback is provided continually in the first week and then is gradually removed in the second week. Run time progressively increases from 15 to 30 min [8,9,11,41,42]. This faded feedback program prevents dependency on external feedback and generates long-term retention [24]. Various other gait retraining programs have been proposed for patients with PFPS [19,49,53]. These include programs using five sessions over eight weeks [19], ten sessions over six weeks [49], and or only one session of ten minutes followed by four weeks of self-administration and monitoring increased step rate [53]. Each has shown improvements in symptoms and function and thus may be considered as an alternative to the above gait retraining prescription. Considerations for type of gait retraining intervention need to account for multiple factors including type of injury being treated, injury duration, time in season, and level of competition, with the goal of developing a long-term strategy to reduce risk for new or recurrent RRI.

Gait retraining implementation must consider the role of muscle strength and fatiguability, as different strategies will have different demands of the neuromotor system. An increase in trunk flexion is associated with greater peak hip extension moment [54], and transition to a forefoot strike is associated with greater peak ankle plantarflexion moment [55]. Strengthening the calf muscles may reduce the incidence of calf soreness that was reported for the step rate [49] and forefoot strike strategies [42]. Similarly, strengthening hip extensor muscles may facilitate a better transition to a gait with increased trunk lean [56]. Further, a combination of gait retraining strategies may be used to achieve the goals of the retraining program. Previous studies based on increasing step rate instructed patients to land softly [49] or to land softly and adopt a non-rearfoot strike pattern if necessary [19].

Regardless of the benefits for injured runners, very limited evidence supports the use of gait retraining for healthy runners. Athletes with VALR greater than 70 BW/s that received visual feedback and were instructed to “run softer” presented a 62% lower occurrence of RRIs in a year [57]. Only one retrospective study provided evidence that RFS runners present higher rates of prior RRIs than non-rearfoot strikers [58] and conversely another cross-sectional study found that a non-RFS was associated with calf muscle injuries and Achilles tendinopathy. Since no prospective studies have been performed, the transition to a forefoot strike in healthy runners cannot be uniformly recommended using evidence-based treatment [59,60]. Finally, limited evidence shows that the transition to a forefoot strike does not change running economy at fast speeds and limited-to-moderate evidence shows a decrease in running economy at low-medium speeds in recreational runners [59]. Therefore, changing the foot strike pattern to improve the running economy is not recommended. The potential of the other strategies to reduce the likelihood of RRIs and improve performance was not assessed.

**Figure 1:**
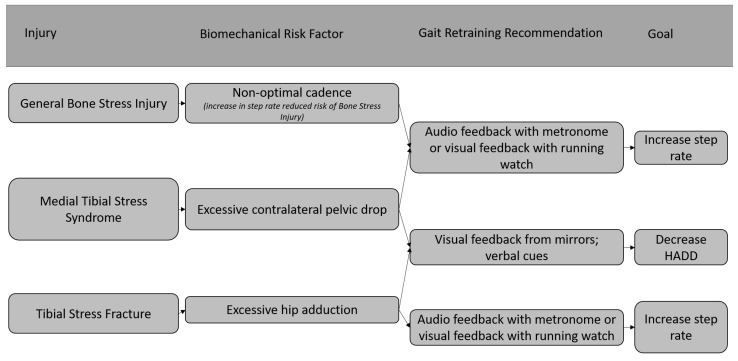
Examples of using gait retraining to treat bone related RRIs [22,30,32,61,62].

**Figure 2:**
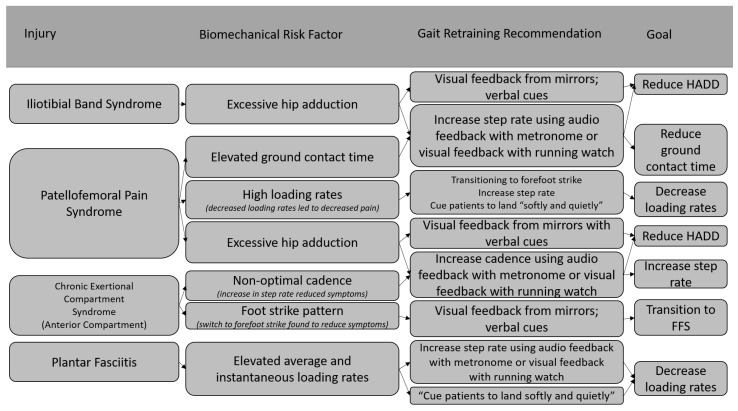
Examples of using gait retraining to treat other specific RRIs [28,29,32,33,34,35,42,46,63,64,65,66,67].

While gait retraining has largely been studied for those with PFPS; limited work has been conducted on addressing biomechanical risk factors in non-PFPS RRI. Examples of gait retraining strategies that may be applied to runners presenting with various injuries are illustrated in Figure 1 and Figure 2. It is important to note that gait retraining has only been used to treat runners with PFPS and chronic exertional compartment syndrome (CECS). Studies examining the effect of gait retraining on pain and functionality in many common running injuries do not currently exist, so specific recommendations for using gait retraining in injured runners with injuries other than PFPS and CECS cannot be made. Figure 1 and Figure 2 serve only to provide examples on how a clinician may treat an injured runner using gait retraining based on literature surrounding gait retraining studies and risk factors for specific RRIs such as medial tibial stress syndrome [30,32], tibial stress fractures [61,62], iliotibial band syndrome [63,64], PFPS [28,29,32,42,65,66,67], CECS [46] and plantar fasciitis [33,34,35].

## 7. Limitations of Current Gait Retraining Strategies

Most studies characterizing gait retraining require participants to limit their running to gait retraining sessions in a laboratory setting [12,14]. This may discourage injured runners with an upcoming race, or those unwilling to take extended time away from running, from undergoing gait retraining. Studies that have allowed participants to continue with their own training schedules outside of gait retraining have found that runners can benefit from performing gait retraining while still participating in training outside of the laboratory [10,16,44]. Gait retraining completed outside of a laboratory setting while still continuing typical training has been observed to significantly reduce footstrike angle and increase step rate [50]. While this study did not report any negative outcomes for participants, RRI risk should be considered when allowing runners to continue with training while completing gait retraining, accounting for time for soft tissue and bone adaptations. Since this study only examined step rate manipulation, adjusting other variables may be less feasible outside of the clinical setting.

Despite the success found in gait retraining strategies employed in many of the studies examined in this review, a study conducted by Esculier et al. [19] found that education of proper training loads and education combined with gait retraining resulted in similar decreases in knee pain in runners with PFPS. Notably the session duration and frequency was lower than standard gait retraining programs and did not use a faded feedback design [8,9,11,14,18,41,42,47]. This discrepancy in results highlights that not all gait retraining protocols are equally effective, and that it is important to identify factors that may aid in the success of a gait retraining protocol.

Presently, it is unknown if gait retraining effectiveness is influenced by severity of injury. For example, improvement in pain and functionality in patients with PFPS following gait retraining only included participants that reported a pain level below a 7 out of 10 on a visual analog scale [42]. A separate investigation on gait retraining instructed PFPS patients to run only when their pain level was below a 2 out of 10 on the visual analog scale [19]. Thus, patients who report severe pain while running may benefit from undergoing other forms of treatment before beginning gait retraining.

There exists a clear gap in the literature surrounding gait retraining in terms of randomized controlled trials. Only two randomized controlled gait retraining studies have examined the effects of gait retraining as a form of injury treatment [19,42]. Both trials examined knee pain as an outcome measure and the two studies found contrasting results. While one study found a significant reduction in knee pain in the group that underwent gait retraining [42], the other study found there was no difference in knee pain in those who had undergone gait retraining and education compared to those who had underwent education alone [19]. Other studies using gait retraining did not record patient pain levels or did not include a control group. No randomized controlled trials have examined the effect of gait retraining on pain and functionality in RRIs other than PFPS. Further investigation into the effects of gait retraining as treatment for specific injuries may reveal that gait retraining is not equally effective for all types of RRIs. Identifying specific injuries that gait retraining is more effective in treating could increase the value of gait retraining as a rehabilitation tool for RRIs.

Only two studies included in this review reported adverse effects [42,49]. Both studies cited soreness of the calves that did not affect the ability of subjects to complete their training. One study reported ankle soreness at a 1-month follow-up after gait retraining. The time to achieve strength and tissue adaption must be individualized to reduce risk for RRI and gait retraining should be progressed gradually. More studies investigating potential adverse effects of gait retraining are needed before gait retraining can be fully recommended as a treatment strategy in injured runners.

## 8. Conclusions

While biomechanical risk factors are variable across RRIs, gait retraining may be used to modify potentially faulty running mechanics. An individualized and diagnosis-specific approach is important to address specific risk factors for the injured runner. Running mechanics can be modified using different forms of biofeedback and should use a faded feedback design for motor learning. Future studies, ideally in a randomized clinical study design, may clarify how different forms of gait retraining may be used, alone or in combination, to treat and prevent RRI.

## Figures and Tables

**Table 1:** Definitions of key biomechanical variables.

Variable Name	Variable Definition
Vertical Impact Peak (VIP)	The local maximum found between initial foot strike and the maximum ground reaction force [12]
Vertical Average Loading Rate (VALR)	Slope of the ground reaction force curve from 20% to 80% of the vertical impact peak, measured in body weights per second (BW/s) [12]
Vertical Instantaneous Loading Rate (VILR)	Maximum slope of the ground reaction force curve from 20% to 80% of the vertical impact peak, measured in BW/s [12]
Braking Impulse	A measure of the total force applied in the posterior direction during stance phase. Area under the anteroposterior ground reaction force curve from initial contact until midstance [36]
Peak Tibial Acceleration	Maximum tibial acceleration at time of initial contact (also known as “impacts”) [12]

**Table 2:** Overview of Gait Retraining Studies.

	Adjusted Variable	Feedback	Subjects	Retraining Design	Outcomes
Noehren et al., 2011 [8]	HADD	Visual display and verbal cues	10 female runners with PFPS and high HADD	Faded, 8 sessions over 2 weeks	86% reduction in pain with 11-point increase in LEFI. Significant reduction in HADD and contralateral pelvic drop. All changes persisted at 1-month follow-up
Willy et al., 2012 [9]	HADD	Visual feedback from mirror and verbal cues	10 female runners with PFPS	Faded, 8 sessions over 2 weeks	Reduced HADD, thigh adduction and contralateral pelvic drop. All changes persisted at the 1- and 3-month follow-ups, although HADD increased from post-trial to 1- and 3-month follow-ups
Esculier et al., 2018 [19]	Step rate	Not clear	69 runners with PFPS	Not faded, 5 sessions over 8 weeks	No difference in KOS-ADLS scores between runners who received both education and gait retraining compared to runners who only received education on load management
Willy et al., 2016 [20]	Step rate	Visual feedback from Garmin Forerunner	30 healthy runners with high loading rates	Faded, 8 runs, no feedback on 4th, 6th or 8th run	Significant increase in step rate, significant reduction in VALR, VILR, HADD and knee eccentric work
Baumgartner et al., 2019 [21]	Step rate	Visual feedback from watch	38 healthy runners, step rate <170	Not faded	Significant increase in step rate from 79.9 +/− 4.8 to 86.8 +/− 5.7 strides per leg per minute
Crowell and Davis 2011 [12]	Tibial acceleration	Visual feedback	10 healthy RFS runners with high tibial acceleration	Faded, 8 sessions over 2 weeks	Significant reductions in tibial acceleration, VALR, VILR that persisted at 1-month follow-up
Clansey et al., 2014 [13]	Tibial acceleration	Visual feedback	22 healthy RFS male runners with high tibial accelerations	Not faded, 6 sessions over 3 weeks	Significant reductions in tibial acceleration, VALR, VILR at post-trial. Only tibial acceleration remained significant at the 1-month follow-up
Bowser et al., 2018 [14]	Tibial acceleration	Visual feedback	19 healthy RFS runners with high tibial acceleration	Faded, 8 sessions over 2 weeks	Significant reductions in tibial acceleration, VIP, VALR, VILR, at follow-up timepoints of 1, 6, and 12 months
Cheung et al., 2018 [39]	Tibial acceleration	Visual feedback	16 healthy runners with high tibial accelerations	Faded, 8 sessions over 2 weeks	In the post-trial participants were distracted but still had significant reduction in VALR, VILR and tibial acceleration compared to pre-trial
Ching et al., 2018 [15]	Tibial acceleration	Audio feedback	16 healthy runners with high tibial acceleration	Faded, 8 sessions over 2 weeks	In the post-trial participants were distracted but still had significant reduction in VALR, VILR and tibial acceleration compared to pre-trial. Additional feedback did not change loading rates in runners that had already undergone gait retraining
Zhang et al., 2019 [17]	Tibial acceleration	Visual feedback	13 healthy runners with high tibial acceleration	Faded, 8 sessions over 2 weeks	37.3% reduction in peak tibial acceleration, runners maintained lower tibial accelerations at +/− 10% of their self-selected pace
Zhang et al., 2019 [16]	Tibial acceleration	Visual feedback	12 healthy runners with high tibial acceleration	Faded, 8 sessions over 2 weeks	Runners were able to maintain lower tibial accelerations during overground running and treadmill slope running, but not overground slope running
Sheerin et al., 2020 [18]	Tibial acceleration	Haptic feedback through watch	18 healthy runners with high tibial acceleration	Faded, 8 sessions over 2 weeks	41% reduction in average tibial acceleration on a treadmill. 17% reduction in tibial acceleration during overground running
da Silva Neto et al., 2022 [40]	Vertical ground reaction force	Visual feedback	24 healthy RFS runners	Not faded, 8 sessions over 2 weeks	Reduced maximum force in the midfoot and medial rearfoot. Showed gait retraining can be performed overground rather than with a treadmill
Cheung and Davis 2011 [41]	Forefoot strike pattern	Audio feedback from buzzer in shoe	3 female runners with PFPS	Faded, 8 sessions over 2 weeks	All 3 participants had decreased VALR and VILR by 10.9–35.1%. Pain scores were improved by 10.4–19.5 points
Roper et al., 2016 [42]	Forefoot strike pattern	Visual feedback from mirror and verbal cues	16 RFS runners with running-related knee pain	Faded, 8 sessions over 2 weeks	Significant reduction in pain from 5.3 to 1.0 at post-trial and 1-month follow-up
Chan et al., 2020 [43]	Midfoot strike pattern	Visual display of footstrike pattern	20 healthy RFS male runners	Faded, 8 sessions over 2 weeks	Only 40% of participants successfully transitioned to midfoot strike pattern, those who did displayed no difference in vertical loading rate
Yang et al., 2020 [44]	Forefoot strike pattern	Audio feedback from mobile app	17 healthy RFS runners	Not faded	Significantly lower loading rates, significantly higher ankle joint moment from pre- to post-study. Significantly lower loading rates in participants who underwent gait retraining and switched to minimalist shoes compared to those who just switched to minimalist shoes
Chan et al., 2021 [45]	Forefoot strike pattern	Audio feedback	16 healthy runners	Faded, 8 sessions over 2 weeks	75% of participants switched to non rearfoot striking over level ground, 94% over uphill running and 88% over downhill running
Teng et al., 2020 [10]	Trunk lean	Visual display of trunk lean	12 healthy RFS runners	Faded, 5 sessions over 8 weeks	Significant reduction in PFJ stress, knee extensor moment, peak ankle plantar flexor moment, significant increase in peak hip extensor moment
Helmhout et al., 2015 [46]	Forefoot strike pattern and step rate	Education and audio feedback from verbal cues	19 military members with chronic extertional compartment syndrome for at least 2 months	Not faded	Significant increase in running distance, significant increase in SANE and LLOS, significant decrease in PSC
Futrell et al., 2020 [47]	Forefoot strike pattern and step rate	Audio feedback from metronome for step rate group, audio feedback for footstrike pattern group	39 healthy RFS runners without a history of bone stress injuries and with step rates below 170	Faded, 8 sessions over 2 weeks	41% reduction in VALR in the footstrike pattern group compared to 14% reduction in VALR in the step rate group at 1-week post-trial. Changes were maintained at 6 months post-trial
Miller et al., 2021 [48]	Forefoot strike pattern and step rate	Audio feedback from metronome and verbal cues	9 injured military service members	Not faded	Significant reduction in VALR, increase in step rate, significant improvement in patient SANE scores. All participants remained injury free at 6-month follow-up
Bonacci et al., 2018 [49]	Footwear and step rate	Audio feedback from metronome	14 RFS runners with PFPS	Faded, 10 sessions over 6 weeks	All subjects in gait retraining had reduction in pain and improvement in function. Significantly lower anterior knee pain compared to orthotics group
Molina-Molina et al., 2022 [50]	Footwear and step rate	Audio feedback from a metronome for step rate group, removal of shoes for barefoot group	70 healthy runners	Not faded, 30 sessions over 3 weeks	Significant decrease in rearfoot strike angle in barefoot group and step rate group. Significant increase in step rate at comfortable speed for step rate group. At a high speed, step rate increased for the barefoot group and decreased for the step rate group.
dos Santos et al., 2019 [11]	Forefoot strike pattern, step rate and forward trunk lean	Audio feedback from clinician for footstrike and forward trunk lean groups, audio feedback from metronome for step rate group	18 runners with PFPS	Faded, 8 sessions over 2 weeks	All 3 groups had decreased pain, increased functionality and decreased LEFS scores from pre- to post-trial. All changes were maintained at a 6-month follow-up. AKPS scores decreased from pre-trial to post-trial in the footstrike and trunk lean groups and between pre-trial and 6-month follow-up in all groups

Clinical Outcomes: LEFI- Lower Extremity Functional Index (same as LEFS), KOS-ADLS- Knee Outcome Survey–Activities of Daily Living Scale, SANE- Single Assessment Numeric Evaluation, LLOS- Lower Leg Outcome Survey, PSC- Patient Specific Complaints questionnaire, LEFS- Lower Extremity Functional Scale, AKPS- Anterior Knee Pain Scale, VAS- Visual Analog Scale.

## Data Availability

Not applicable.
